# Innovative vaginal manipulator technique vs. traditional method for vaginal fornix deployment in robotic sacrocolpopexy

**DOI:** 10.3389/fsurg.2024.1491233

**Published:** 2024-11-07

**Authors:** Yoshiaki Ota, Kuniaki Ota, Toshifumi Takahashi, Shogo Kawamura, Mitsuru Shiota, Koichiro Shimoya

**Affiliations:** ^1^ Department of Obstetrics and Gynecology, Kawasaki Medical School, Okayama, Japan; ^2^ Fukushima Medical Center for Children and Women, Fukushima Medical University, Fukushima, Japan

**Keywords:** pelvic organ prolapse, pelvic organ prolapse quantification, robotic sacrocolpopexy, spatula, vaginal manipulator, vaginal fornix

## Abstract

**Introduction and hypothesis:**

Pelvic organ prolapse (POP) affects approximately 30% of middle-aged and older women, with 11%–19% requiring surgical intervention. Laparoscopic sacrocolpopexy preserves the vaginal axis and length but involves a steep learning curve and longer operation times. Robotic sacrocolpopexy (RSC) addresses these issues, offering enhanced surgical precision. This study aimed to evaluate the feasibility and effectiveness of a novel vaginal manipulator (Hoyte Sacro Tip®; Cooper Surgical, Trumbull, CT, USA) compared with the traditional spatula in RSC.

**Methods:**

This retrospective cohort study included 88 females undergoing RSC at Kawasaki Medical School Hospital between January 2021 and December 2023. Patients were divided into two groups: spatula (*n* = 50) and vaginal manipulator (*n* = 38). Data on patient demographics, operative outcomes, and postoperative POP quantification (POP-Q) scores were collected.

**Results:**

Baseline characteristics were similar between the groups, except for gravidity and hypertension, which were higher in the spatula group than that in the vaginal manipulator group. No significant differences were found in operative time, console time, estimated blood loss, or complication rates between the groups (*p* = 0.08, 0.12, 0.19, and NA, respectively). Hospital stays were shorter in the vaginal manipulator group (median 6.5 vs. 7.0 days, *p* = 0.03) than in the spatula group. Both groups showed improved POP-Q scores postoperatively. However, the vaginal manipulator group had significantly lower ΔC scores than that of the spatula group (6.26 ± 3.88 vs. 8.53 ± 3.25, *p* = 0.02).

**Conclusions:**

The vaginal manipulator proved to be a safe and feasible alternative to the traditional spatula, with comparable perioperative outcomes and shorter hospital stays. The manipulator's design facilitated better tissue dissection, potentially improving surgical efficiency.

## 1 Introduction

Pelvic organ prolapse (POP) is a condition in which organs in the pelvis, such as the uterus, bladder, or rectum, descend from their normal position and bulge into the vagina. Approximately 30% of middle-aged and older women experience some degree of prolapse (1), and approximately 11%–19% of women with POP require surgery (2).

Sacrocolpopexy can preserve the normal vaginal axis and maximum vaginal length by securing the vaginal apex to the anterior surface of the sacrum (3, 4). While laparoscopic sacrocolpopexy (LSC) offers faster recovery times and reduced blood loss, it typically takes longer to perform than an open approach. Additionally, surgeons require a steeper learning curve to master the laparoscopic technique (5).

Robotic sacrocolpopexy (RSC) has been investigated as a solution to these problems. The three-dimensional view, increased freedom of movement, ability to perform sophisticated suturing, and ease of knot-tying provided by robotic instruments are the ultimate advantages of RSC. Therefore, numerous clinical centers have switched from LSC to RSC.

**Figure 1:**
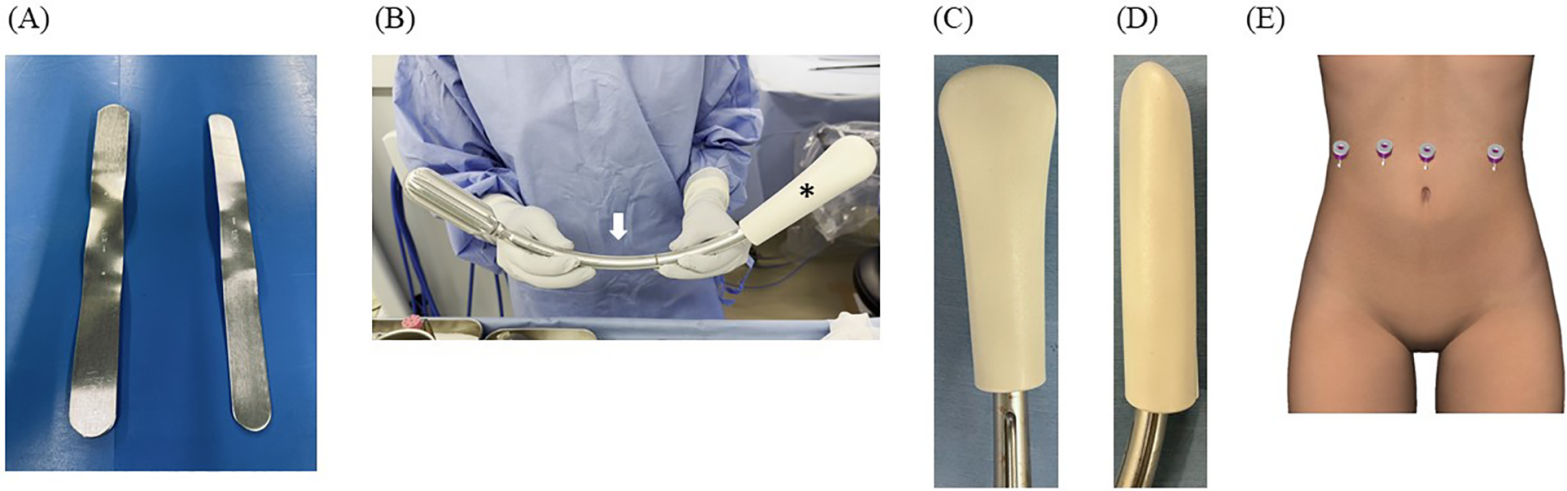
**(A)** The spatula on the left is 35 mm and on the right is 30 mm in width. **(B)** The vaginal manipulator (Hoyte Sacro Tip®, Cooper Surgical, Shelton, CT) consists of a reusable manipulator handle (white arrow) and a disposable sacrocolpopexy tip (black asterisk). **(C** and **D)** The Hyote Sacro Tip® is constructed in a uniquely thick dome shape. **(E)** Port placement for robotic-assisted sacrocolpopexy using the da Vinci Xi surgical system (Intuitive Surgical Inc., Sunnyvale, CA, USA).

Both LSC and RSC demonstrate similar hospital stays, recovery times, anatomical outcomes, pelvic floor function, and quality of life improvements compared to open surgery (6–8). While RSC may have longer operative times than LSC, this difference can diminish with increased surgical experience (6). Ultimately, the choice between approaches depends on surgeon expertise, hospital resources, and patient-specific factors, with minimally invasive techniques (laparoscopic or robotic) generally preferred when feasible due to their reduced morbidity and faster recovery times (7, 8). On the other hand, LSC and RSC limit adequate tissue exposure and weaken the mesh placement owing to a lack of tactile feedback and disadvantages of a limited number of degrees of freedom compared to abdominal sacrocolpopexy (9). In particular, the degree of repair of anterior compartment descensus is lower in LSC than in open surgery (10). To compensate for these disadvantages, the traditional spatula (Figure 1A) helps to create space between the vagina and other organs; however, a new vaginal manipulator (Hoyte Sacro Tip®; Cooper Surgical, Trumbull, CT, USA) has been specifically designed for this task (Figures 1B–D), which may potentially improve mesh placement.

Although the new vaginal manipulator for RSC offers potential advantages, its effectiveness compared with the traditional spatula remains unclear owing to a lack of clinical outcome data. This study aimed to verify the feasibility and effectiveness of the new vaginal manipulator by primarily comparing POP quantification (POP-Q) scores following RSC with the conventional spatula method, and secondary comparing surgical outcomes between the two approaches as well.

## 2 Materials and methods

### 2.1 Study design and ethical approval

This was a retrospective cohort study. The study was conducted in accordance with the Declaration of Helsinki. The study was approved by the Institutional Review Board of Kawasaki Medical School (protocol code: 5070-013; no. 5043-03). Informed consent was replaced by an opt-out method, which enabled patients to voluntarily participate or withdraw from the study.

### 2.2 Patient recruitment

Patients with minimally invasive sacrocolpopexy performed between January 2021 and December 2023 at the Kawasaki Medical School hospital were recruited into this study. We retrospectively reviewed the medical records of patients who underwent RSC with the spatula: 50 patients (January 2021–July 2022) and those who underwent RSC with the vaginal manipulator: 38 patients (August 2022–December 2023). Inclusion criteria were patients who underwent RSC with symptomatic vaginal vault prolapse or descensus uteri and POP-Q stage ≥2. Exclusion criteria were: poor health status [severe obesity (body mass index >35 kg/m^2^), heart failure (NYHA class III-IV) (11), and stage III-IV of chronic obstructive pulmonary disease (12)] with inability to undergo general anesthesia, age <18 years, ≥3 laparotomic surgeries, and known pelvic malignancies. POP surgery was only performed if the patient was symptomatic.

### 2.3 Data acquisition

Medical records were reviewed to collect data, including age; body mass index; gravidity and parity; detailed gynecologic, medical, and surgical histories; POP-Q stage; type of POP (cystocele, uterine prolapse, rectocele, cystocele + uterine prolapse, cystocele + rectocele, uterine prolapse + rectocele, and cystocele + uterine prolapse + rectocele); and operative outcomes, including operation and console times, estimated blood loss, operative complications, and the number of days of hospital stay. Operation time was defined as the time from skin incision to the completion of RSC and concomitant procedures, and console time was defined as the time for performing the procedure at the console from the beginning of RSC to the closure of the retroperitoneum with the placement of an adhesion barrier. The timing of each intracorporeal surgical procedure was rechecked by reviewing recorded surgical videos.

### 2.4 Surgical techniques for RSC

After inducing general anesthesia, the patient was positioned in a lithotomy position with the Trendelenburg position. All robotic procedures were performed using the robotic four-arm Da Vinci Xi surgical system (Intuitive Surgical Inc., Sunnyvale, CA, USA). A 12-mm robotic port for the camera scope was inserted by direct puncture 2 cm above the umbilicus to start a pneumoperitoneum, and three 8-mm robotic ports were placed on the same horizontal line of the umbilicus, 7 cm apart (Figure 1E). The instruments were set with the fenestrated bipolar on the first arm, the Maryland bipolar or Mega needle driver with shear scissors on the third arm, and Cadière forceps on the fourth arm. The Maryland bipolar was set at 70 W to prepare for cutting and coagulation. A hysterectomy was performed if the patient requested it.

#### 2.4.1 Sacral promontory exposure

RSC began with sacral promontory exposure. An incision was made from the medial side of the right common iliac artery to expose the fifth lumbar vertebra dorsal to the hypogastric nerve and the first sacral vertebra ventral to the anterior longitudinal ligament using a bipolar cut. A retroperitoneal tunnel was then formed from the right side of the rectum to the medial side of the ureter near the uterosacral ligament while preserving the hypogastric nerve.

#### 2.4.2 Dissection of the anterior and posterior vaginal fornices

**Figure 2:**
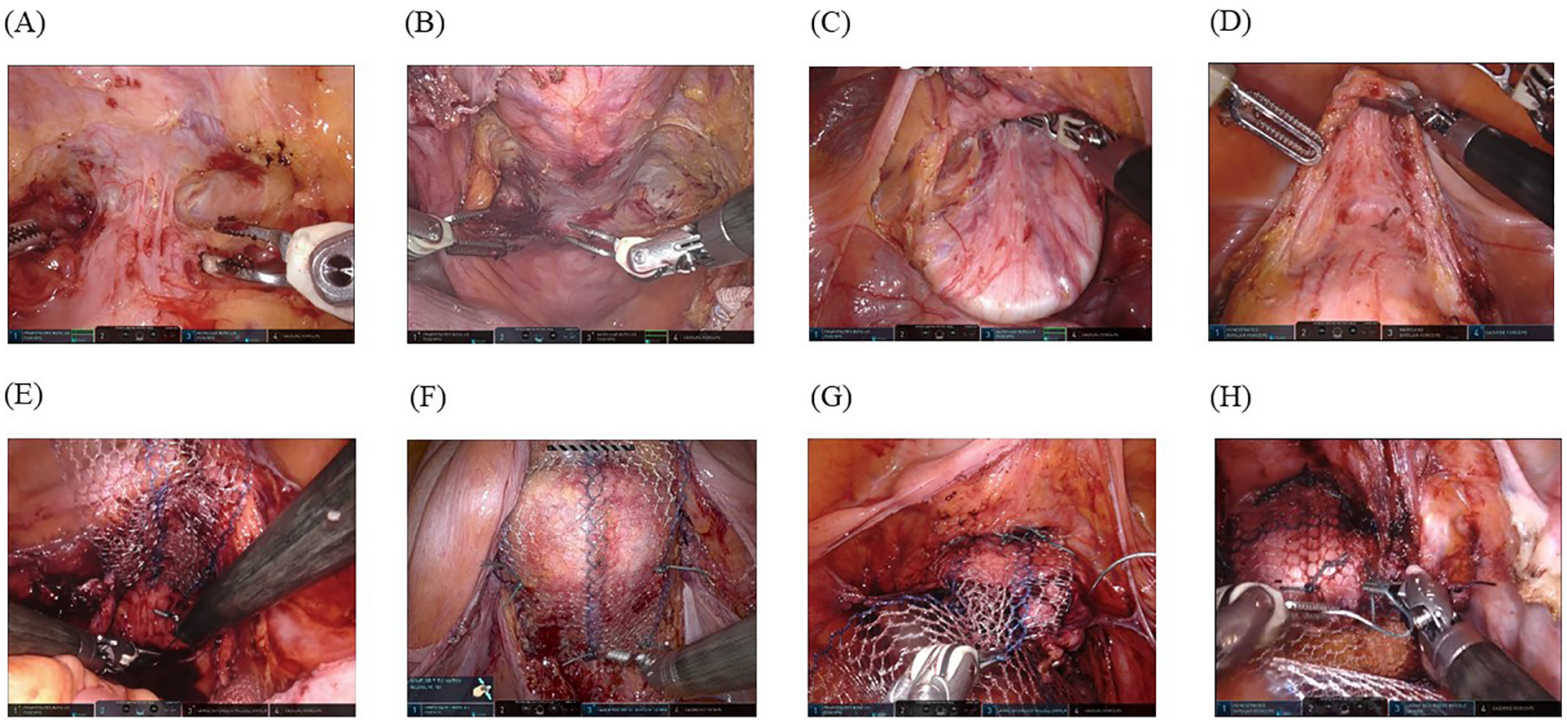
Surgical techniques: **(A)** A spatula is firmly placed in the posterior fornix to facilitate its exposure. **(B)** A vaginal manipulator (Hoyte Sacro Tip®) is even better for exposing the posterior fornix owing to its dome shape. **(C)** A spatula is firmly placed in the anterior fornix to facilitate its exposure, and the plane between the bladder and vaginal wall is thus opened using careful dissection with a bipolar in-cut mode using the Maryland forceps, followed by dissection of the anterior vaginal fornix to point Aa (3 cm from the external urethral orifice). **(D)** A vaginal manipulator (Hoyte Sacro Tip®) is even better at exposing the anterior fornix with the same technique because it is suitable for the space of the anterior vaginal fornix to point Aa owing to the domed shape. For the space exposed by the spatula **(E)** and vaginal manipulator **(F)**, the posterior self-cut polypropylene mesh is fixed with a nonabsorbable suture to levator ani fascia bilaterally. For the anterior vaginal wall exposed by the spatula **(G)** and vaginal manipulator **(H)**, the mesh is firmly fixed with six sutures.

The spatula (Figure 2A) or vaginal manipulator (Figure 2B) was firmly placed in the posterior fornix to facilitate its exposure. The pouch of Douglas was opened by making an incision on the peritoneum, and the rectovaginal space and levator ani fascia were reached bilaterally. In addition, the spatula (Figure 2C) or vaginal manipulator (Figure 2D) was also firmly placed in the anterior fornix to facilitate its exposure. Thus, the plane between the bladder and the vaginal wall was opened by careful dissection using the bipolar cut mode of the Maryland forceps, and subsequently, the anterior vaginal fornix was dissected to point Aa (3 cm from the external urethral orifice) (spatula, Figure 2C; vaginal manipulator, Figure 2D).

#### 2.4.3 Hysterectomy

Once the anterior and posterior vaginal fornices were exposed, the round and suspensory ligaments of the ovary were incised with the Maryland forceps in the third arm. The vesicouterine pouch and the pouch of Douglas were opened vaginally, and the uterus was excised by severing the uterine parenchyma from the sacrouterine ligament. Vaginal cuff closure was performed using absorbable interrupted sutures (0-vicryl®; Ethicon, Inc., Somerville, NJ, USA) with CT-1 needles after successful removal of the extirpated uterus from the vagina.

#### 2.4.4 Mesh placement

A previously configured posterior self-cut polypropylene-mesh (Gynemesh®; Ethicon, Inc.) was inserted through the third port by an assistant and fixed with a nonabsorbable suture (#0-SH ETHIBOND EXCEL®; Ethicon, Inc.) to the levator ani fascia bilaterally (spatula, Figure 2E; vaginal manipulator, Figure 2F). Thereafter, we fixed the posterior vaginal fornix with three sutures to prevent wrinkle formation within the meshes. When the exposure was complete in the posterior vaginal fornix, the anterior self-cut polypropylene-mesh (Gynemesh®; Ethicon, Inc.) was inserted through the third port by an assistant, and two nonabsorbable running sutures (#0-SH ETHIBOND EXCEL®; Ethicon, Inc.) were placed to fix the mesh with six stitches to the anterior vaginal wall (spatula, Figure 2G; vaginal manipulator, Figure 2H). Both tails of the anterior and posterior meshes were sewn together, passed through the retroperitoneal tunnel, and fixed to the sacral promontory using two nonabsorbable sutures, avoiding excessive tension on the mesh. The retroperitoneal space was closed using continuous absorbable sutures (Vicryl 2-0®; Ethicon, Inc.). Subsequently, a sheet of Seprafilm® (KAKEN PHARMACEUTICAL CO., LTD., Tokyo, Japan) was cut into four pieces, inserted from the third port by an assistant, and placed with robot arms onto the surgical sites to prevent adhesion.

### 2.5 Evaluation of surgical outcomes

Significant intraoperative complications (organ injury or hemorrhage) and early postoperative complications (hemorrhage, infection, or early reoperation before hospital discharge) were also recorded. All patients had a postoperative physical examination for the evaluation of pelvic floor disorders. POP was postoperatively classified according to the POP-Q system (13) to confirm the evaluation of the delta (*Δ*), which was obtained by subtracting postoperative scores from preoperative scores to determine whether postoperative anatomy improved.

### 2.6 Statistical analysis

Statistical analyses were performed using EZR (Saitama Medical Center, Jichi Medical University, Saitama, Japan), a graphical user interface for R (The R Foundation for Statistical Computing, Vienna, Austria) (14). The one-sample Kolmogorov–Smirnov test was used to test the abnormal distribution of the quantitative data. Data were represented as the median and interquartile range for non-parametric variables, and categorical variables were described as frequency and percentage and compared between the groups using the Mann–Whitney U test (for numeric non-parametric variables) or Fisher's exact test (for categorical variables). RSC procedures with the spatula and vaginal manipulator were compared using the Pearson's chi-square, Fisher's exact, or Student's *t*-test, as appropriate. All tests were two-sided and were considered statistically significant at a level of 0.05.

## 3 Results

The baseline characteristics of the study population are shown in Table 1. A total of 88 females who underwent RSC were included in the study (spatula, *n* = 50; vaginal manipulator, *n* = 38). There were no significant differences in the baseline characteristics between the groups, except for gravidity and hypertension. Gravidity was significantly higher in the spatula group than that in the vaginal manipulator group (median 3.0, 2.0–4.0 vs. median 2.5, 2.0–3.0; *p* = 0.03). The percentage of patients with hypertension was significantly higher in the spatula group than that in the vaginal manipulator group (54.0% vs. 28.9%, *p* = 0.03).

**Table 1:** Baseline clinical characteristics between spatula and vaginal manipulator groups.

	Spatula group (*n* = 50)	Vaginal manipulator group (*n* = 38)	*P*-value
Age at surgery, years	71.5 (66.0–76.2)	72.0 (65.5–75.8)	0.91
Body mass index, kg/m^2^	25.1 (22.9–27.3)	23.3 (20.6–26.1)	0.06
Gravidity	3.0 (2.0–4.0)	2.5 (2.0–3.0)	0.03
Parity	3.0 (2.0–3.0)	2.0 (2.0–4.0)	0.07
Hypertension (%)	27 (54.0)	11 (28.9)	0.03
Diabetes mellitus (%)	10 (10.0)	4 (10.5)	0.26
Tobacco use (%)	6 (12.0)	1 (2.6)	0.23
Previous surgery			
Hysterectomy (%)	1 (2.0)	2 (5.3)	0.58
Caesarian sections (%)	0 (0.0)	3 (7.9)	0.08
Pelvic surgeries (%)	9 (18.0)	7 (18.4)	1.00

Data are presented as median (interquartile range).

The preoperative characteristics of the patients with POP in the spatula and vaginal manipulator groups are summarized in Table 2. All patients ranked in at least the second degree in the POP-Q stage. The POP-Q stage was significantly different between the spatula and vaginal manipulator groups (*p* < 0.01). There were no significant differences in the percentages of types of POP between the spatula and vaginal manipulator groups (*p* = 0.15).

**Table 2:** Preoperative characteristics of the patients with pelvic organ prolapse between the spatula and vaginal manipulator groups.

	Spatula group (*n* = 50)	Vaginal manipulator group (*n* = 38)	*P*-value
POP-Q stages			
Stage II (%)	29 (58.0)	3 (7.9)	<0.01
Stage III (%)	19 (38.0)	27 (71.1)	
Stage IV (%)	2 (4.0)	8 (21.0)	
The types of POP			
Cystocele (%)	16 (32.1)	12 (31.6)	0.15
Uterine prolapse (%)	13 (26.0)	3 (7.9)	
Rectocele (%)	5 (10.0)	3 (7.9)	
Cystocele + uterine prolapse (%)	7 (14.0)	4 (10.5)	
Cystocele + rectocele (%)	5 (10.0)	8 (21.1)	
Uterine prolapse + rectocele (%)	0	1 (2.6)	
Cystocele + uterine prolapse + rectocele (%)	4 (8.0)	7 (18.4)	

POP-Q, pelvic organ prolapse quantification; POP, pelvic organ prolapse.

Perioperative outcomes of the patients with POP in the spatula and vaginal manipulator groups are shown in Table 3. No statistically significant differences were found in operation time, console time, estimated blood loss, and complication rate. The length of hospital stay in the spatula group was significantly longer than that in the vaginal manipulator group (median 7.0, 6.0–7.8 vs. median 6.5, 6.0–7.0; *p* = 0.03).

**Table 3:** Comparison of perioperative outcomes between the spatula and vaginal manipulator groups.

	Spatula group (*n* = 50)	Vaginal manipulator group (*n* = 38)	*P*-value
Operation time, min	164.5 (150.3–180.0)	174.0 (159.3–189.0)	0.08
Console time, min	133.0 (117.3–153.5)	148.0 (126.3–153.0)	0.12
Estimated blood loss, ml	30.0 (10.0–50.0)	4.5 (23.5–73.8)	0.19
Complication (%)	0 (0)	0 (0)	NA
Hospital stays, day	7.0 (6.0–7.8)	6.5 (6.0–7.0)	0.03

Data are presented as median (interquartile range). NA, not applicable.

Table 4 summarizes the preoperative and postoperative POP-Q scores for both the spatula and vaginal manipulator groups. Postoperative POP-Q scores for Aa, Ba, C, Ap, and Bp showed improvement compared to the corresponding preoperative scores in both groups. However, the preoperative and postoperative POP-Q scores for pb and Gh did not show significant changes in either group. In the vaginal manipulator group, there was no change in the total vaginal length (TVL) POP-Q score before and after surgery, whereas in the spatula group, the TVL score improved postoperatively compared with preoperatively.

**Table 4:** Preoperative and postoperative measurement of POP-Q scores in both the spatula and vaginal manipulator groups.

	Spatula group (*n* = 50)		*P*-value	Vaginal manipulator group (*n* = 38)		*P*-value
	Pre	Post		Pre	Post	
POP-Q, cm						
Aa	+1 (–1 to +2)	–3 (–3 to –3)	<0.01	+3 (+2 to +3)	–3 (–3 to –3)	<0.01
Ba	+1 (–1 to +2)	–3 (–3 to –3)	<0.01	+3 (+2 to +3)	–3 (–3 to –3)	<0.01
C	0 (–4 to +2)	–7 (–8 to –7)	<0.01	0 (–3 to +3)	–6 (–7 to –5)	<0.01
Ap	+1 (–1 to +2)	–3 (–3 to –3)	<0.01	+3 (+2 to +3)	–3 (–3 to –3)	<0.01
Bp	–1.8 (–2.0 to +1.0)	–3 (–3 to –3)	<0.01	0 (–1.4 to +2.0)	–3 (–3 to –3)	<0.01
pb	+3 (+3 to +4)	3 (+3 to +4)	0.57	+3 (+3 to +5)	+3 (+3 to +5)	1.00
Gh	+4 (+4 to +4.5)	4 (+4 to +4.5)	0.10	+4 (+4 to +4)	+4 (+4 to +4)	0.49
TVL	+9 (+8 to +9)	+8 (+7 to +9)	<0.01	+7 (+7 to +8)	+7 (+7 to +8)	1.00

Data are presented as median (interquartile range). POP-Q, pelvic organ prolapse quantification; TVL, total vaginal length.

**Figure 3:**
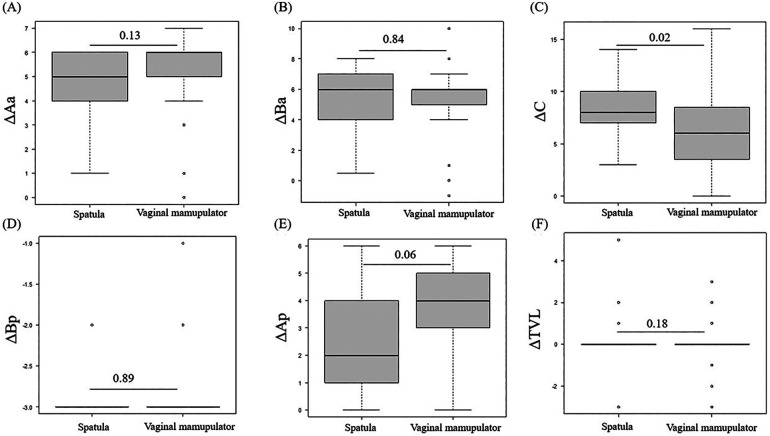
The comparison of ΔAa **(A)**, ΔBa **(B)**, ΔC **(C)**, ΔBp **(D)**, ΔAp **(E)**, and ΔTVL **(F)** between the spatula and vaginal manipulator groups at POP-Q stages III and IV. POP-Q, pelvic organ prolapse quantification; TVL, total vaginal length.

Figures 3A–F summarizes the comparison of ΔAa, ΔBa, ΔC, ΔBp, ΔAp, and ΔTVL (the difference between preoperative and postoperative POP-Q scores at each site) between the spatula and vaginal manipulator groups at POP-Q stages III and IV. Owing to a bias in POP-Q stage distribution between the spatula and vaginal manipulator groups, with more than half (58%) of the spatula group in POP-Q stage II, comparisons before and after surgery were limited to POP-Q stages III and IV. There were no significant differences in ΔAa, ΔBa, ΔBp, ΔAp, and ΔTVL between the two groups. Only the ΔC in the vaginal manipulator group was significantly lower than that in the spatula group (*p* = 0.02).

## 4 Discussion

This study confirmed that RSC performed using the novel vaginal manipulator instead of a spatula is a safe and feasible minimally invasive treatment for POP repair, with satisfactory anatomic postoperative results.

Parity is a leading risk factor for POP in rodents and primates, significantly reducing tangent modulus and vaginal tensile strength in animal models (15). Consequently, the vaginal wall in patients with POP becomes hyperextensible, necessitating firm dissection of the septum between the vagina and bladder or rectum for non-recurrent sacrocolpopexy. Restoring apical support to the vagina is crucial for long-term anatomical success, as approximately 50% of anterior prolapse is due to apical support loss (16–18). Ensuring apical support during hysterectomy for POP also reduces recurrence and reoperation rates (19). Therefore, the dissection plane must be clearly exposed to securely hold the mesh.

**Figure 4:**
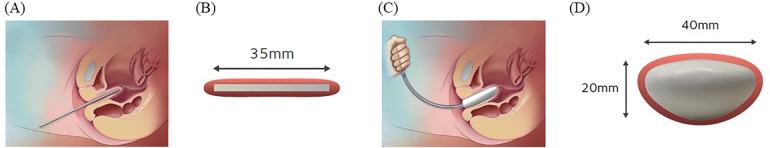
In sagittal **(A)** and axial **(B)** images of spatula insertion into the vaginal canal, the spatula is limited in opening the space owing to its flat shape. In sagittal **(C)** and axial **(D)** images of vaginal manipulator insertion, the ability of the manipulator to facilitate the vaginal wall separation from the bladder and rectum, owing to its dome shape, is shown.

The originally used spatula is flat, causing uneven pressure on the vaginal wall and insufficient vaginal canal spread, leading to inadequate dissection (Figures 4A,B). In contrast, the vaginal manipulator (Hoyte Sacro Tip®, Cooper Surgical), a thick, dome-shaped vaginal manipulator with a contoured tip, fits securely and comfortably in the vagina. It facilitates the separation of the vaginal wall from the bladder and rectum, providing a streamlined suturing surface (Figures 4C,D). Jeanditgautier et al. reported that larger meshes decrease pelvic organ mobility, enhancing support and reducing descent (20). The vaginal manipulator can be firmly placed in the fornix to fully expose the larger plane with appropriate counteraction. Instrument selection for insertion into the vagina in RSC is challenging; however, the dome-shaped tip of the vaginal manipulator significantly aids this process. Consequently, we achieved surgical results comparable with the conventional method.

The da Vinci Surgical System (Intuitive Surgical Inc.) provides three-dimensional vision and wristed instruments with six degrees of freedom, enhancing complex dissection and suturing and addressing the limitations of the laparoscopic-only approach. Robotic techniques for POP repair have increased, with a meta-analysis showing lower blood loss and abdominal conversion rates in RSC compared with LSC, despite longer operative times for RSC (21). Reducing operating time in RSC is crucial owing to additional docking and console time. Techniques requiring tactile perception in confined spaces are particularly challenging in endoscopic surgeries such as RSC and LSC. Minor technical improvements can potentially shorten the operating time. Although this pilot study indicated that the new vaginal manipulator was not inferior to conventional methods, deeper dissection may enhance pelvic organ elevation and reduce operative time. Future studies with multiple surgeons are needed to quantify the ease of surgery between vaginal manipulators and conventional methods.

The synthetic polypropylene mesh implant has been the most commonly used for pelivc organ prolapse due to its easy availability and affordability, although there are concerns regarding risks of vaginal pain, reoperation for mesh exposure, and lack of evidence that mesh results in better subjective outcomes (22, 23). Following the post-market surveillance by the Food and Drug Administration (FDA), the National Institute for Health and Care Excellence, and the FDA to ban polypropylene mesh for, not RSC or LSC, only transvaginal mesh (TVM) in the UK and USA, amongst other countries which have followed suit (24). Currently, mesh erosion has been described to be the most frequent and severe complication, and it often takes three years or more for such symptoms to arise, hence the importance of clinical trials lasting more than three years to be fully assessed to balance the pros and cons of the surgery (25). On the other hand, mesh exposure is low following all modes of sacrocolpopexy (open, robotic, and laparoscopic) in the survey for a median of 6.5 years (26). In particular, the incidence rate of mesh erosion following robotic was lowest compared to open and laparoscopic [open 7.7% (95% CI 4.6–12.5%); robotic 3.6% (95% CI 1.7%–7.6%); laparoscopic 4.9% (95% CI 3.1%–7.7%); *p* = 0.20] (26). In this study, polypropylene mesh was used, and POP-Q was evaluated before and after surgery, but long-term complications, particularly mesh erosion, were not analyzed, so long-term follow-up at least three years is planned for the future.

This study had several limitations. First, it was a single-center retrospective study rather than a multicenter randomized controlled trial, potentially limiting its generalizability. Second, the study had a relatively small sample size, and the use of the vaginal manipulator in RSC was recent, possibly affecting surgeon proficiency compared with the spatula. The most recent RSC cases using the vaginal manipulator were advanced POP cases based on preoperative POP-Q assessments. Third, the relatively short-term follow-up data precluded a comparison of long-term effectiveness and safety between the spatula and vaginal manipulator. However, the study also had strengths. First, it was a comparative analysis of the spatula and vaginal manipulator based on data from consecutive procedures performed by a single surgeon, minimizing variability in surgical skills. Second, the study employed a validated POP-Q system, both preoperatively and postoperatively. In addition, when comparing the spatula and vaginal manipulator before and after RSC, it seemed that the spatula is superior at POP-Q scores for C. However, this significant difference is not thought to have any clinical significance, because the symptoms of the patients have improved in both the spatula and vaginal manipulator. In other words, this study has shown that surgical procedures comparable to the traditional method using the spatula can be performed using the innovative vaginal manipulator we have introduced.

In conclusion, RSC with the new vaginal manipulator had comparable perioperative outcomes to RSC with the conventional spatula. The dome-shaped tip of the new vaginal manipulator allows for easier dissection of the vaginal wall compared with the conventional method. In the future, RSC using the vaginal manipulator should be performed in multiple centers to study the ease of surgery and long-term prognosis. Furthermore, although RSC has been defined as a minimally invasive surgery, recently, sacrocolpopexy using a percutaneous system, which is even more minimally invasive, has been reported (27, 28). In the future, we will need to try using a vaginal manipulator in sacrocolpopexy with a percutaneous system and challenge even more minimally invasive surgery.

## Data Availability

The original contributions presented in the study are included in the article/Supplementary Material, further inquiries can be directed to the corresponding author.
